# Regional Variation in Brain White Matter Diffusion Index Changes following Chemoradiotherapy: A Prospective Study Using Tract-Based Spatial Statistics

**DOI:** 10.1371/journal.pone.0057768

**Published:** 2013-03-04

**Authors:** Christopher H. Chapman, Mohammad Nazem-Zadeh, Oliver E. Lee, Matthew J. Schipper, Christina I. Tsien, Theodore S. Lawrence, Yue Cao

**Affiliations:** 1 Department of Radiation Oncology, University of Michigan, Ann Arbor, Michigan, United States of America; 2 Department of Biostatistics, University of Michigan, Ann Arbor, Michigan, United States of America; 3 Department of Radiology, University of Michigan, Ann Arbor, Michigan, United States of America; 4 Department of Biomedical Engineering, University of Michigan, Ann Arbor, Michigan, United States of America; University of Maryland, College Park, United States of America

## Abstract

**Purpose:**

There is little known about how brain white matter structures differ in their response to radiation, which may have implications for radiation-induced neurocognitive impairment. We used diffusion tensor imaging (DTI) to examine regional variation in white matter changes following chemoradiotherapy.

**Methods:**

Fourteen patients receiving two or three weeks of whole-brain radiation therapy (RT) ± chemotherapy underwent DTI pre-RT, at end-RT, and one month post-RT. Three diffusion indices were measured: fractional anisotropy (FA), radial diffusivity (RD), and axial diffusivity (AD). We determined significant individual voxel changes of diffusion indices using tract-based spatial statistics, and mean changes of the indices within fourteen white matter structures of interest.

**Results:**

Voxels of significant FA decreases and RD increases were seen in all structures (p<0.05), with the largest changes (20–50%) in the fornix, cingula, and corpus callosum. There were highly significant between-structure differences in pre-RT to end-RT mean FA changes (*p*<0.001). The inferior cingula had a mean FA decrease from pre-RT to end-RT significantly greater than 11 of the 13 other structures (*p*<0.00385).

**Conclusions:**

Brain white matter structures varied greatly in their response to chemoradiotherapy as measured by DTI changes. Changes in FA and RD related to white matter demyelination were prominent in the cingula and fornix, structures relevant to radiation-induced neurocognitive impairment. Future research should evaluate DTI as a predictive biomarker of brain chemoradiotherapy adverse effects.

## Introduction

Radiation therapy for brain neoplasms–with or without chemotherapy–is limited by toxicity to healthy brain tissue, which is not easily predicted by dose alone [1]. One major symptom is neurocognitive impairment, a delayed and irreversible effect characterized by selective declines in short-term memory and attention [2]–[6]. While this toxicity is thought to be a result of many interacting processes, it is known that brain white matter, composed of axonal fibers and their myelin sheaths, is particularly sensitive to radiation [7]. Radiation therapy-induced white matter degradation has been associated with neurocognitive impairment in animals and humans [2], [8]. However, non-invasive anatomical studies in humans have been difficult in the past, because white matter degradation may not be visible on conventional magnetic resonance imaging (MRI) [2], [9].

Diffusion tensor imaging (DTI) is a non-invasive MRI-based technique that is more sensitive to white matter structure than conventional MRI [10]. DTI measures water molecule diffusion in the brain, which varies with the direction, density, and myelination of white matter fibers. Two derived indices of this measurement are radial diffusivity (RD) and axial diffusivity (AD), which are associated with the magnitudes of diffusion perpendicular and parallel to white matter fibers respectively. Increased RD has been correlated to decreased myelination in several models of brain injury, including radiation-induced demyelination [11]–[15]. Decreased AD has been correlated to axonal degeneration and radiation-induced reactive astrogliosis [11], [14]–[16]. Another commonly used diffusion index is fractional anisotropy (FA), a normalized index which ranges from zero (equal diffusion in all directions) to one (diffusion along a single axis only). FA reflects overall white matter density and integrity, and decreased FA has been seen in many brain pathologies [10]. The first uses of DTI to assess chemoradiotherapy effects were in pediatric patients, where a reduction in FA was found without white matter lesions on conventional MRI [17], [18]. Along with preclinical data, this demonstrates that DTI is sensitive to treatment-induced white matter degradation.

DTI’s sensitivity to white matter degradation can be used to determine whether there is regional variation in the toxic effects of radiation therapy within the brain. This is important because the brain is currently considered a homogeneous structure for radiation dose-volume calculations and toxicity prediction, but it is not known whether this is accurate [19]. Identifying structures of particular sensitivity to radiation could support proposals of targeted dose-sparing in order to prevent neurocognitive impairment [20], [21]. The majority of current dose-sparing proposals have focused on protecting the hippocampus, including an ongoing phase II clinical trial [22]. Evidence that radiation impairs hippocampal neural stem cell differentiation has advanced the hypothesis that this is the primary mechanism behind late-delayed cognitive decline [23]. Support for neural stem cell-sparing radiation techniques makes the presumption that preserving neurogenesis alone will prevent neurocognitive impairment. However, if the associated white matter is damaged, then the networks that produce neurocognitive functions are not intact. Our approach is to determine whether preferential damage to any white matter structures may also contribute to neurocognitive impairment. Previous studies have compared diffusion indices in pediatric brain radiation patients to controls, and found lower FA in frontal lobe, temporal lobe, and periventricular white matter, demonstrating localized white matter damage [18], [24], [25]. Longitudinal studies have looked at diffusion index changes in a few selected regions [26]–[30], but there has not yet been a study which has systematically analyzed longitudinal diffusion index changes in whole brain white matter. This information could impartially identify sensitive white matter structures, which would be valuable for developing radiation dose-sparing techniques and imaging biomarkers.

The current study aimed to (i) examine regional variation in white matter chemoradiotherapy sensitivity throughout the whole brain, and (ii) investigate changes in diffusion indices which are influenced by distinct pathological processes. To accomplish this, we obtained diffusion tensor images from brain metastasis patients before and after whole-brain radiation and chemotherapy. Maps of the diffusion indices FA, RD, and AD were calculated for each patient at each time point. Using tract-based spatial statistics (TBSS), we evaluated individual voxel changes in these indices with automated white matter tract finding [31]. We then partitioned these results into volumes representing the major white matter structures in the brain, in order to analyze diffusion index changes within these structures over time. Our final analysis evaluated differences between structures in the magnitude of diffusion index changes, in order to identify regional variation in white matter chemoradiotherapy sensitivity. Completion of these aims is important for better understanding the pathogenesis of treatment-induced toxicity, and designing techniques for predicting and preventing this outcome.

## Materials and Methods

### Ethics Statement

A prospective imaging study with human subjects was approved by the Institutional Review Boards of the University of Michigan Medical School (Federal Wide Assurance #00004969). Written informed consent was obtained for all participants.

### Study Design and MRI

Patients with cancer metastatic to the brain received fractionated whole-brain radiation. Radiation was delivered in opposed lateral fields to 30 Gy in 3 Gy daily fractions or 37.5 Gy in 2.5 Gy daily fractions. Treatment dose was calculated at the point of maximum field separation. Patients underwent MRI scans at three time points: within two weeks before starting RT (pre-RT), within one week after finishing RT (end-RT), and one month after finishing RT (one month post-RT). All images were obtained on a 3T Achieva scanner (Phillips, The Netherlands). MRI sequences included diffusion tensor imaging (DTI), T1- and T2-weighted images. DTI was acquired using a spin-echo echo-planar imaging sequence with a repetition time of 7032 ms, echo time of 62 ms, 224×224×120 mm^3^ field of view, 128×128 matrix, and 1.75×1.75×2.0 mm^3^ voxels with no gap between slices. For each axial slice, diffusion sensitizing gradient encoding with a diffusion weighting factor of *b* = 1000 s/mm^2^ was applied in 15 non-collinear directions, plus one null image with *b* = 0 s/mm^2^.

### Image Pre-processing and Diffusion Tensor Calculation

All image pre-processing before TBSS analysis was done with FIAT (Functional Imaging Analysis Tool), an in house software package [32]. All diffusion weighted images were registered to the null image of the same series using mutual information and rigid body transformation. Diffusion tensors were then computed at each voxel and eigenvalues were determined, from which three diffusion index maps were generated: axial diffusivity (AD), radial diffusivity (RD), and fractional anisotropy (FA).

### Image Selection Criteria

**Figure 1:**
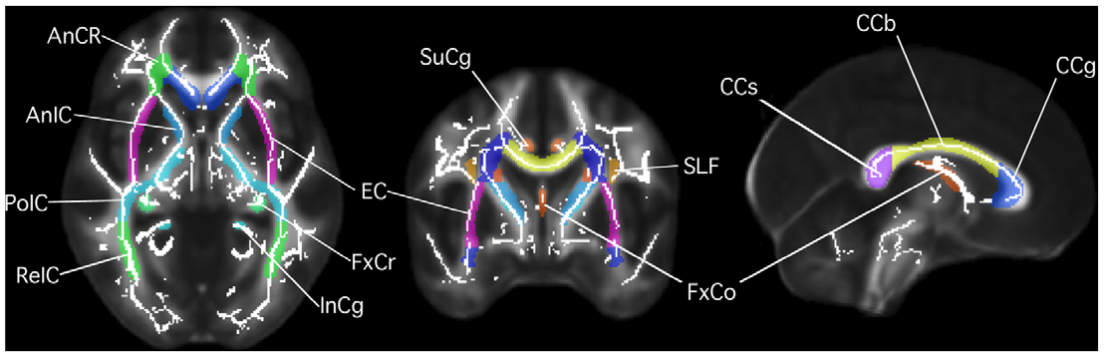
White matter regions of interest. ICBM DTI-81 white matter atlas [33] depicted on FMRIB58 standard-space FA map [35]. Data-derived white matter skeleton mask in white. AnCr, anterior corona radiata; AnIC, anterior internal capsules; PoIC, posterior internal capsules; ReIC, retrolenticular internal capsules; EC, external capsules; FxCr, fornix crura; InCg, inferior cingula; SuCg, superior cingula; SLF, superior longitudinal fasciculi; FxCo, fornix columns; CCs, corpus callosum splenium; CCb, corpus callosum body; CCg, corpus callosum genu. Posterior corona radiata not depicted.

Before analysis, fourteen white matter structures representing the major supratentorial white matter tracts of the brain were selected from the International Consortium of Brain Mapping (ICBM) DTI-81 white matter atlas, which is registered to the ICBM-152 standard-space anatomical template (Table 1; Figure 1) [33]. To choose patients with major white matter tracts free from metastasis influence, FIAT was used to compare the non-diffusion weighted images to the white matter atlas structures by the following steps. All T1- and T2-weighted images were interpolated to 1 mm^3^ voxels. T1-weighted images were registered to the ICBM-152 anatomical template by affine transformation [34]. The same transformation matrix was then used to deform the T2-weighted images. Masks of the fourteen white matter structure volumes were overlaid on the template-registered T1- and T2-weighted images, and any patients with non-punctate metastases (defined as >5 mm diameter), edema, or mass effect in the structures at any time point were excluded from further analysis. Punctate metastases (≤5 mm diameter) were permitted in white matter structures if there was no surrounding edema or interval change on T1- and T2-weighted images. The original non-deformed images were used to visually confirm that the structures were free from tumor influence.

**Table 1:** Fourteen white matter structures.

White Matter Structure	Paired
Corpus Callosum Body	No
Corpus Callosum Genu	No
Corpus Callosum Splenium	No
Cingula, Inferior	Yes
Cingula, Superior	Yes
Corona Radiata, Anterior	Yes
Corona Radiata, Posterior	Yes
External Capsules	Yes
Fornix Columns	No
Fornix Crura	Yes
Internal Capsules, Anterior	Yes
Internal Capsules, Posterior	Yes
Internal Capsules, Retrolenticular	Yes
Superior Longitudinal Fasiculi	Yes

### Computation of Image Skeletons

The FMRIB Software Library (FSL) was used for the following image processing steps previously explained in detail [31], [35]. Briefly, FA maps from all patients and time points were interpolated to 1 mm^3^ voxels and registered to the FMRIB58 standard-space FA image by nonlinear transformation. This target was chosen instead of the “most typical” subject method [31] to minimize potential registration error due to metastases. These co-registered FA maps were averaged to produce a mean FA map. On this mean FA map, the local direction of greatest FA slope was found around each voxel, defining the direction perpendicular to the local white matter tract at each voxel. The voxels representing the local FA maximum along these directions were labeled, producing a one-voxel thick “skeleton” near the center of white matter tracts. The volume of this mean FA skeleton above threshold FA = 0.2 was then used to create a skeleton mask, in order to exclude minor tracts, non-white matter voxels, and areas of possible misalignment. The skeleton mask was projected using the same voxel assignments back to the original individual FA maps to confirm correct registration of white matter tracts. FA maps for each patient and all time points were then projected onto the skeleton mask by assigning the maximum FA value along the direction perpendicular to the skeleton mask (defined above) to the nearest skeleton mask voxel. This created a one-to-one voxel correlation between all subjects’ FA maps, allowing for individual voxel statistics. The same nonlinear transformation matrices and perpendicular projections were also applied to all AD and RD maps to generate skeletonized forms of these maps as well.

### Tract-based Spatial Statistics

Tract-based spatial statistics were performed using FSL. Significant differences in FA, AD, and RD values between all time points (pre-RT to end-RT, pre-RT to one month post-RT, end-RT to one month post-RT) were determined by subject-paired two-sided *t*-tests. Maps of *t* values were augmented with threshold-free cluster enhancement to reduce noise [36], and correction for multiple comparisons was achieved using a nonparametric permutation test with 5000 permutations [37], Significance level was set at α = 0.05. At this point in analysis, all white matter skeleton voxels were used, including those outside the fourteen white matter structures defined above.

### Within-Structure Analysis

We identified fourteen volumes of the skeleton mask representing the white matter structures of interest. These were labeled as the spatial intersection between the corresponding ICBM DTI-81 white matter atlas structure and the skeleton mask, with visual confirmation of appropriate fit. Mean values for FA, AD, and RD within each skeletonized structure of interest for all patients and time points were calculated from the voxel values. The longitudinal changes of mean diffusion index values within each structure of interest were calculated for all patients and time intervals (pre-RT to end-RT, pre-RT to one month post-RT, end-RT to one month post-RT). Statistical analysis consisted of two steps. First, significant left-right asymmetries of diffusion index longitudinal changes were determined by subject-paired two-sided *t*-tests. Paired structures were combined where significant asymmetry of diffusion index longitudinal changes did not exist. Second, the significance of the longitudinal changes themselves was determined by subject-paired two-sided *t*-tests. At both steps, corrections for multiple comparisons were performed by a Bonferroni-type sequential procedure to control false discovery rate. A comparison was considered statistically significant when *p_i_*<α*_i_* where *p_i_* is the *i* ^th^ smallest *p*-value from *m* comparisons and α*_i_* = 0.05*i*/*m* [38].

### Between-Structure Group Analysis

Change in FA from pre-RT to end-RT was chosen as a representative diffusion index and time interval. Mean FA changes were compared between structures using repeated-measures ANOVA in two steps. First, repeated-measures ANOVA was performed with different structures representing the within-subject factor and different patients representing the between-subject factor. Additional between-subject dichotomous factors were evaluated: patient age (above and below median age), radiation dose (30 Gy and 37.5 Gy), and sex (male and female). Levene’s test and Mauchly’s test were used to assess homoscedasticity and sphericity, assumptions of repeated-measures ANOVA. Violation of sphericity was corrected using the Greenhouse-Geisser method to reduce type I error. Significance level was set at α = 0.05. Second, post hoc comparisons were performed using subject-paired two-sided *t*-tests to compare mean FA change in the structure with the greatest change to changes in all other structures (thirteen comparisons). For these post hoc comparisons, Bonferroni correction was used with α = 0.05/13 ≈ 0.00385.

## Results

### Study Completion and Image Selection

Of thirty-one enrolled patients who underwent imaging at pre-RT, fourteen underwent imaging at end-RT and were free from metastasis influence in the white matter regions of interest, permitting inclusion for analysis (Table 2). Ten of the fourteen included patients had one or fewer non-punctate metastases. These were fairly evenly distributed throughout the brain across patients, minimizing the average effect of tumor influence in any single brain area. Nine patients received bortezomib concurrent with RT on a separate clinical trial, 1.7 mg/m^2^ every 72 hours for four doses. Three other patients (7, 10, and 13) received one cycle of chemotherapy between end-RT and one month post-RT. Two patients did not complete imaging at one month post-RT and were excluded from analysis at that time point.

**Table 2:** Patient characteristics.

No.	Sex	Age(y)	Dose(Gy)	Chemotherapy	Primary Cancer	Metastases(>5 mm)	Location (lobe)	Scans
1	Male	40	37.5	Bortezomib	Melanoma	2	Left Frontal, Left Occipital	2/3
2	Female	60	37.5	None	Non-small cell lung	0	–	2/3
3	Male	41	37.5	Bortezomib	Melanoma	1	Left Thalamus	3/3
4	Female	52	37.5	Bortezomib	Melanoma	1	Left Parietal	3/3
5	Male	49	30	Bortezomib	Melanoma	2	Left Temporal, Left Temporal	3/3
6	Female	45	30	Bortezomib	Melanoma	1	Right Frontal	3/3
7	Male	52	30	Paclitaxel &Carboplatin	Non-small cell lung	0	-	3/3
8	Female	55	30	Bortezomib	Melanoma	1	Right Frontal	3/3
9	Male	76	30	Bortezomib	Melanoma	1	Left Frontal	3/3
10	Female	64	37.5	Gemcitabine	Non-small cell lung	0	–	3/3
11	Female	57	30	Bortezomib	Melanoma	2	Right Thalamus, Right Occipital	3/3
12	Male	60	30	Bortezomib	Melanoma	2	Left Cerebellum, Right Temporal	3/3
13	Male	43	30	Cetuximab	Squamous cell, tonsil	0	–	3/3
14	Female	66	37.5	None	Non-small cell lung	1	Left Frontal	3/3

### TBSS Voxel Analysis

**Figure 2:**
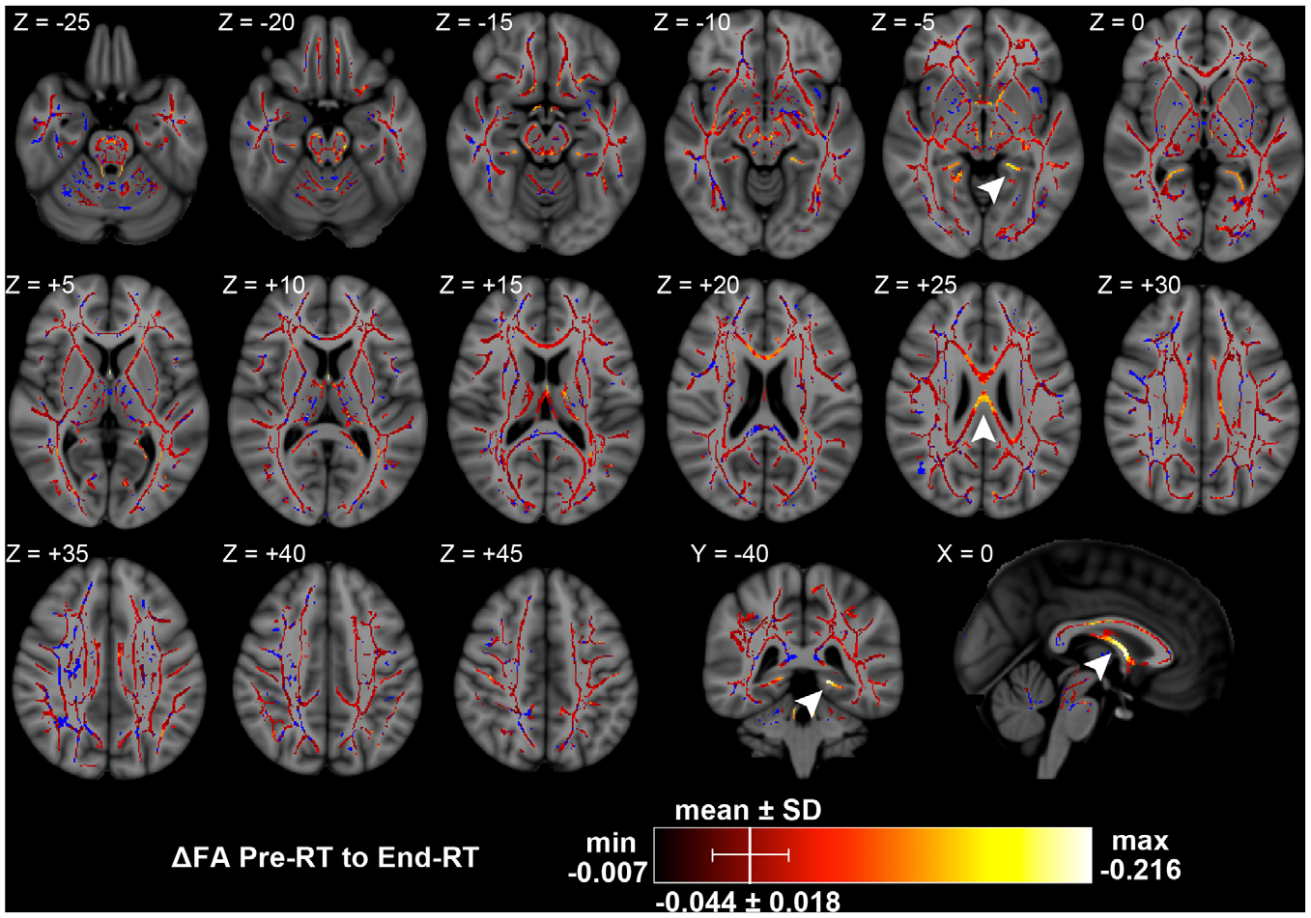
Significant changes in FA from pre-RT to end-RT. Arrowheads: Z = −5, left inferior cingulum; Z = +25, corpus callosum body; Y = −40, left inferior cingulum; X = 0, fornix columns. Significant results per color chart, blue is TBSS skeleton without significant results. Depicted on Montreal Neurological Institute (MNI) ICBM152 standard brain T1-weighted image [34].

The white matter skeleton used for individual voxel statistics consisted of 112,388 voxels of 1 mm^3^. From pre-RT to end-RT, statistically significant FA decreases were seen in 87.8% of white matter skeleton voxels, representing widespread treatment-induced changes. Of voxels with significant FA decreases, the minimum was –0.007 (–3.1%), the maximum was –0.216 (–34.4%), and the mean was –0.044 with a standard deviation of 0.018 (–9.7±4.2%), a skewed distribution towards larger FA decreases. These larger FA decreases were clustered in the columns of the fornix at approximately –0.200 (–30%), in the left inferior cingulum at approximately –0.180 (–30%), and in the posterior corpus callosum body at approximately –0.130 (–20%) (Figure 2).

**Figure 3:**
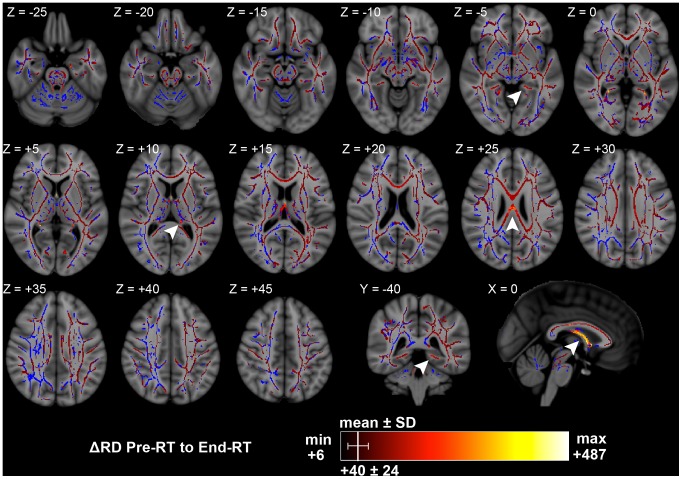
Significant changes in RD from pre-RT to end-RT. Arrowheads: Z = −5, left inferior cingulum; Z = +10, left fornix crus; Z = +25, corpus callosum body; Y = −40, left inferior cingulum; X = 0, fornix columns. Significant results per color chart, blue is TBSS skeleton without significant results. Depicted on MNI ICBM152 standard brain T1-weighted image [34]. Units are µm^2^/s.

There were significant RD increases from pre-RT to end-RT in 64.6% of white matter skeleton voxels, also representing treatment-induced changes. Of voxels with significant RD increases, the minimum was +6 µm^2^/s (+1.5%), the maximum was +487 µm^2^/s (+86.6%), and the mean was +40 µm^2^/s with a standard deviation of 24 µm^2^/s (+10.0±6.7%), also a skewed distribution towards larger RD increases. These larger RD increases were clustered in the columns and crura of the fornix at approximately +300 µm^2^/s (+50%), in the left and right inferior cingulum at approximately +250 µm^2^/s (+40%), and the posterior corpus callosum body at approximately +175 µm^2^/s (+45%) (Figure 3). These clusters of large RD increases were very similar in distribution to the large FA decreases. Of all white matter skeleton voxels, 63.1% of white matter skeleton voxels showed significant changes in both FA and RD. Nearly all areas showing significant RD increases also showed significant FA increases.

**Figure 4:**
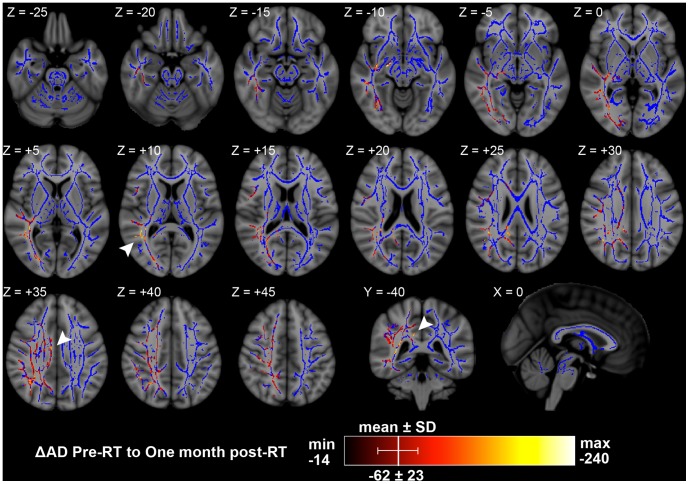
Significant changes in AD from pre-RT to one month post-RT. Arrowheads: Z = +10, right superior longitudinal fasciculus; Z = +35, right superior cingulum; Y = −40, right superior cingulum. Significant results per color chart, blue is TBSS skeleton without significant results. Depicted on MNI ICBM152 standard brain T1-weighted image [34]. Units are µm^2^/s.

There were no significant changes in AD between pre-RT and end-RT. However, between pre-RT and one month post-RT there were significant AD decreases in 12.9% of white matter skeleton voxels. Of voxels with significant AD decreases, the minimum was –14 µm^2^/s (–1.7%), the maximum was –240 µm^2^/s (–18.7%), and the mean was –62 µm^2^/s with a standard deviation of –23 µm^2^/s (–6.4±2.3%), a skewed distribution towards larger AD decreases. These larger AD decreases were clustered in the right superior longitudinal fasciculus at approximately –200 µm^2^/s (–18%), and in the right superior cingulum at approximately –120 µm^2^/s (–12%) (Figure 4). Significant AD changes were seen at different locations and time intervals than significant RD changes.

**Figure 5:**
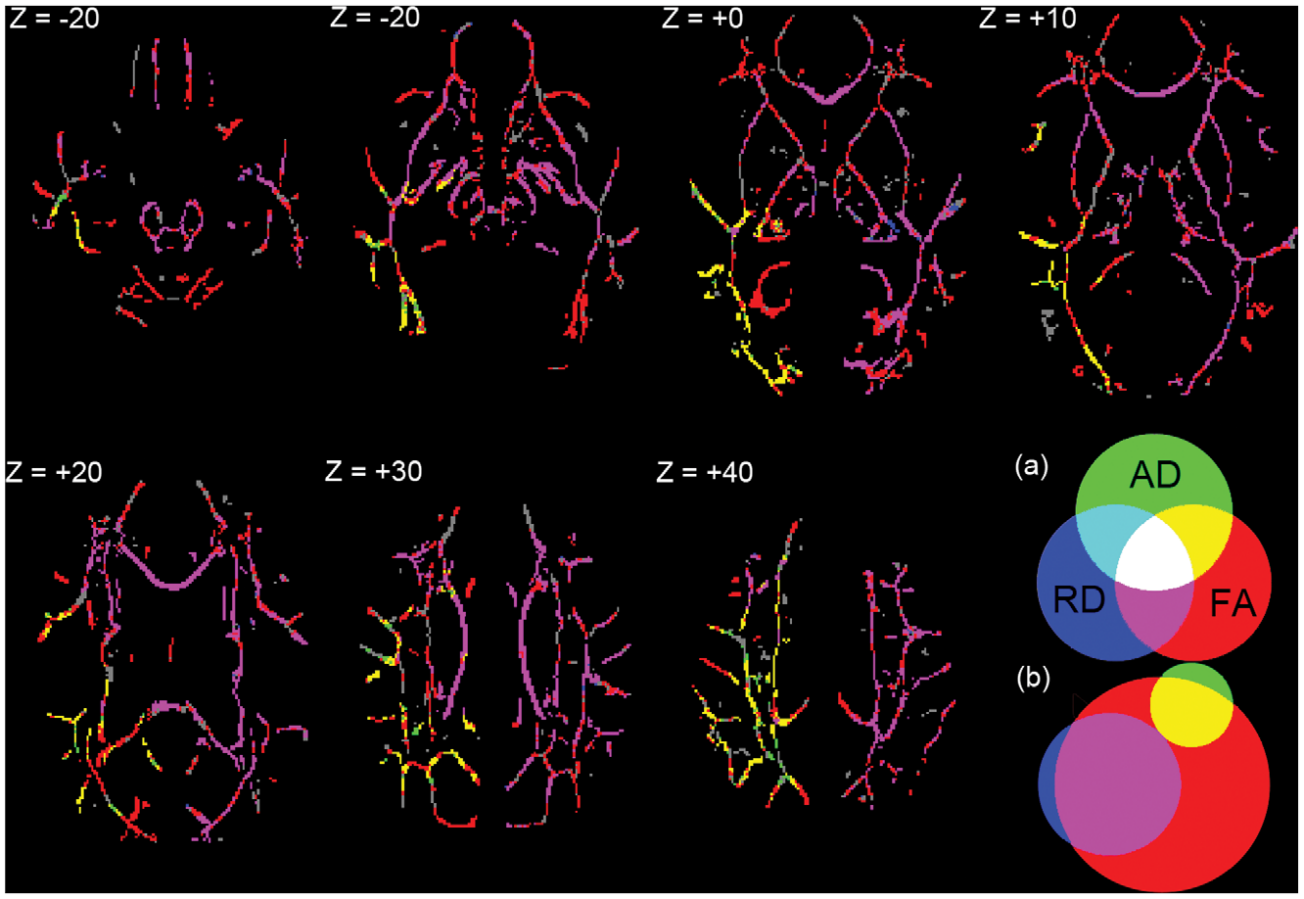
Pre-RT to one month post-RT significant voxel overlap. (a) Color key to significant voxel changes from pre-RT to one month post-RT in AD, RD, and FA with overlapping voxels. Underlying white matter skeleton with no significant changes is gray. (b) Venn diagram depicting proportion of white matter skeleton with significant changes. FA = 82.9%; RD = 37.4%; AD = 12.9%; FA ∩ RD = 33.5%; FA ∩ AD = 10.4%; RD ∩ AD = 0.1%. Labels are MNI coordinates.

From pre-RT to one month post-RT, there continued to be significant FA decreases in 82.9% of white matter skeleton voxels, with clusters of relatively large FA decreases in the same regions as seen from pre-RT to end-RT (Figure S1). From pre-RT to one month post-RT, there continued to be relatively large RD increases in the same regions as before, however only 37.4% of white matter skeleton voxels showed significant increases in RD, a large reduction from the fraction showing significant RD increases from pre-RT to end-RT (64.6%). There were fewer voxels showing significant RD increases in the peripheral white matter (Figure S2), suggesting partial resolution of an RD specific process by one month after radiation. Between pre-RT and one month post-RT, 33.5% of white matter skeleton voxels showed significant changes in both FA and RD, and 10.4% of the skeleton voxels showed significant changes in both FA and AD, indicating large spatial overlap between FA and the other two diffusion indices. Only 0.1% of the skeleton voxels showed significant changes in both AD and RD between pre-RT and one month post-RT, indicating virtually no spatial overlap between AD and RD changes (Figure 5). From end-RT to one month post-RT there were no significant voxel changes in RD, AD, or FA.

### Within-Structure Analysis

Of the total white matter skeleton, 91,222 voxels (81.2%) were labeled as one of fourteen structures representing the major supratentorial white matter tracts. The ten laterally paired structures (Table 1) did not show any statistically significant left-right asymmetry in mean FA, AD, or RD changes between any time points. Therefore, longitudinal analysis was performed using the mean values from combined paired structures.

From pre-RT to end-RT, all fourteen structures showed statistically significant mean FA decreases and mean RD increases (Table 3, Table 4). Similar to the individual voxel results, the largest mean FA decreases (< –0.05 µm^2^/s) from pre-RT to end-RT were seen in the inferior and superior cingula, corpus callosum body and genu, and fornix columns and crura. The largest mean RD increases (> +50 µm^2^/s) from pre-RT to end-RT were in the fornix columns and crura, inferior cingula, and corpus callosum body and genu. Also similar to the voxel results, the superior cingula and superior longitudinal fasciculi showed significant decreases in mean AD from pre-RT to one month post-RT. However, these structures also showed significant mean AD decreases from pre-RT to end-RT, unlike the voxel results and possibly due to averaging of individual voxel changes within these structures (Table 5).

**Table 3:** Fractional anisotropy (FA) longitudinal changes by structure.

		Pre-RT to End-RT			Pre-RT to One Month Post-RT			End-RT to One Month Post-RT	
White Matter Structure	Pre-RT FA	Mean ΔFA	95% CI	Rank	Mean ΔFA	95% CI	Rank	Mean ΔFA	95% CI
Corpus Callosum Body	0.701	**–0.066**	**±0.014**	**2**	**–0.070**	**±0.016**	**2**	*–0.007*	*±0.012*
Corpus Callosum Genu	0.711	**–0.051**	**±0.016**	**6**	**–0.057**	**±0.018**	**3**	*–0.008*	*±0.014*
Corpus Callosum Splenium	0.781	**–0.034**	**±0.014**	**13**	**–0.038**	**±0.015**	**10**	*–0.007*	*±0.009*
Cingula, Inferior	0.559	**–0.085**	**±0.017**	**1**	**–0.078**	**±0.022**	**1**	*+0.003*	*±0.020*
Cingula, Superior	0.597	**–0.052**	**±0.015**	**5**	**–0.050**	**±0.009**	**6**	*–0.002*	*±0.011*
Corona Radiata, Anterior	0.457	**–0.036**	**±0.008**	**11**	**–0.035**	**±0.008**	**12**	*+0.000*	*±0.006*
Corona Radiata, Posterior	0.495	**–0.036**	**±0.008**	**12**	**–0.037**	**±0.010**	**11**	*–0.001*	*±0.006*
External Capsules	0.453	**–0.045**	**±0.012**	**8**	**–0.045**	**±0.010**	**7**	*–0.004*	*±0.008*
Fornix Columns	0.333	**–0.054**	**±0.021**	**4**	**–0.051**	**±0.018**	**5**	*–0.002*	*±0.012*
Fornix Crura	0.531	**–0.054**	**±0.010**	**3**	**–0.052**	**±0.021**	**4**	*+0.000*	*±0.018*
Internal Capsules, Anterior	0.585	**–0.043**	**±0.013**	**9**	**–0.039**	**±0.015**	**9**	*–0.001*	*±0.009*
Internal Capsules, Posterior	0.685	**–0.034**	**±0.016**	**14**	**–0.030**	**±0.014**	**14**	*+0.003*	*±0.022*
Internal Capsules, Retrolenticular	0.601	**–0.046**	**±0.012**	**7**	**–0.043**	**±0.017**	**8**	*+0.001*	*±0.015*
Superior Longitudinal Fasiculi	0.505	**–0.037**	**±0.012**	**10**	**–0.033**	**±0.010**	**13**	*+0.002*	*±0.012*

Statistically significant changes after correction for false discovery rate bolded; non-significant italicized.

**Table 4:** Radial diffusivity (RD) longitudinal changes by structure.

		Pre-RT to End-RT			Pre-RT to One Month Post-RT			End-RT to One Month Post-RT	
White Matter Structure	Pre-RT RD	Mean ΔRD	95% CI	Rank	Mean ΔRD	95% CI	Rank	Mean ΔRD	95% CI
Corpus Callosum Body	323	**+71**	**±17**	**2**	**+71**	**±18**	**1**	*+1*	*±13*
Corpus Callosum Genu	314	**+55**	**±20**	**4**	**+55**	**±23**	**3**	*+2*	*±15*
Corpus Callosum Splenium	252	**+32**	**±20**	**8**	**+33**	**±25**	**6**	*+4*	*±11*
Cingula, Inferior	357	**+67**	**±24**	**3**	**+52**	**±23**	**4**	*–8*	*±17*
Cingula, Superior	370	**+34**	**±13**	**7**	**+27**	**±11**	**8**	*–4*	*±9*
Corona Radiata, Anterior	458	**+28**	**±11**	**10**	**+21**	**±11**	**13**	*–7*	*±8*
Corona Radiata, Posterior	438	**+26**	**±18**	**12**	**+23**	**±18**	**10**	*–3*	*±12*
External Capsules	446	**+28**	**±14**	**11**	**+26**	**±14**	**9**	*+0*	*±9*
Fornix Columns	211	**+112**	**±38**	**1**	*+60*	*±73*	*2*	*–48*	*±58*
Fornix Crura	437	**+51**	**±11**	**5**	**+45**	**±19**	**5**	*–3*	*±18*
Internal Capsules, Anterior	359	**+29**	**±18**	**9**	**+22**	**±18**	**11**	*–5*	*±14*
Internal Capsules, Posterior	308	**+25**	**±14**	**13**	**+22**	**±15**	**12**	*–2*	*±20*
Internal Capsules, Retrolenticular	396	**+36**	**±14**	**6**	**+30**	**±23**	**7**	*–4*	*±18*
Superior Longitudinal Fasiculi	432	**+24**	**±12**	**14**	*+13*	*±14*	*14*	*–11*	*±12*

Statistically significant changes after correction for false discovery rate bolded; non-significant italicized.

Units are µm^2^/s.

**Table 5:** Axial diffusivity (AD) longitudinal changes by structure.

		Pre-RT to End-RT		Pre-RT to One MonthPost-RT		End-RT to One Month Post-RT	
Structure	Pre-RT AD	Mean ΔAD	95% CI	Mean ΔAD	95% CI	Mean ΔAD	95% CI
Corpus Callosum Body	1287	*+8*	*±23*	*–12*	*±19*	**–23**	**±19**
Corpus Callosum Genu	1275	*+13*	*±30*	*–9*	*±27*	*–24*	*±22*
Corpus Callosum Splenium	1317	*–5*	*±30*	*–21*	*±35*	*–17*	*±16*
Cingula, Inferior	908	*+8*	*±35*	*–4*	*±41*	*–3*	*±18*
Cingula, Superior	1048	**–40**	**±20**	**–54**	**±30**	*–15*	*±17*
Corona Radiata, Anterior	954	*–3*	*±15*	*–15*	*±14*	*–12*	*±14*
Corona Radiata, Posterior	985	*–9*	*±15*	*–15*	*±25*	*–7*	*±16*
External Capsules	927	*–16*	*±15*	*–22*	*±2*	*–7*	*±18*
Fornix Columns	1996	*+32*	*±61*	*–35*	*±91*	**–89**	**±70**
Fornix Crura	1028	*+3*	*±18*	*–10*	*±28*	*–6*	*±18*
Internal Capsules, Anterior	1006	*–11*	*±24*	*–18*	*±24*	*–5*	*±19*
Internal Capsules, Posterior	1108	*–13*	*±16*	*–19*	*±24*	*–8*	*±23*
Internal Capsules, Retrolenticular	1135	*–20*	*±21*	*–30*	*±26*	*–12*	*±19*
Superior Longitudinal Fasiculi	977	**–22**	**±16**	**–37**	**±27**	*–17*	*±18*

Statistically significant changes after correction for false discovery rate bolded; non-significant italicized.

Units are µm^2^/s.

From pre-RT to one month post-RT, there were again significant mean FA decreases in all structures, greatest in the inferior cingula, corpus callosum body and genu, and fornix columns and crura (Table 3). Again similar to the voxel results, there were fewer structures showing significant mean RD increases from pre-RT to one month post-RT as compared to pre-RT to end-RT. Notably, the fornix columns no longer showed a significant change despite having the second highest mean RD increase from pre-RT to one month post-RT, due to a wide variation in this change across patients and large confidence interval. Of the structures showing a significant mean RD increase between pre-RT and one month post-RT, the greatest changes were in the corpus callosum body and genu, inferior cingula, and fornix crura (Table 4).

Between end-RT to one month post-RT, there were no significant changes seen in FA and RD, again similar to the voxel results. However, there were significant AD decreases seen in the corpus callosum body and in the fornix columns between end-RT and one month post-RT, despite no significant AD changes in these structures from pre-RT to end-RT or to one month post-RT (Table 5). This was unlike the voxel results, and may again be due to the averaging of individual voxel changes within these structures.

### Between-Structure Analysis

Repeated-measures ANOVA revealed a highly significant (*p*<0.001) difference between structures in pre-RT to end-RT FA changes following chemoradiotherapy (Table 6). Levene’s test of equality revealed no significant differences in error variance between patients for all structures (all *p*>0.05), indicating applicability of ANOVA. Mauchly’s test of sphericity revealed a significant departure from sphericity across structures (*p*<0.001), therefore degrees of freedom for estimating within-subject effects were adjusted using the Greenhouse-Geisser method with ε = 0.311. For within-subject and between-subject variance, there were no statistically significant contributions by age, sex, or radiation dose.

**Table 6:** Repeated-measures ANOVA of FA changes from pre-RT to end-RT.

Within-Subject Effects				
Source	Type III Sum of Squares	Degrees of Freedom	F-value	*P*-value
Structure	0.036	4.127	8.831	<0.001
Structure * Dose	0.004	4.127	1.102	0.369
Structure * Age	0.002	4.127	0.512	0.732
Structure * Sex	0.001	4.127	0.344	0.852
Error	0.041	41.271	–	–
**Between-Subject Effects**				
**Source**	**Type III Sum of Squares**	**Degrees of Freedom**	**F-value**	***P*** **-value**
Intercept	0.452	1	108.798	<0.001
Dose	0.002	1	0.542	0.478
Age	0.007	1	1.799	0.210
Sex	<0.001	1	0.006	0.942
Error	0.042	10	–	–

Degrees of freedom for within-subject effects corrected for lack of sphericity by Greenhouse-Geisser method, ε = 0.311.

**Figure 6:**
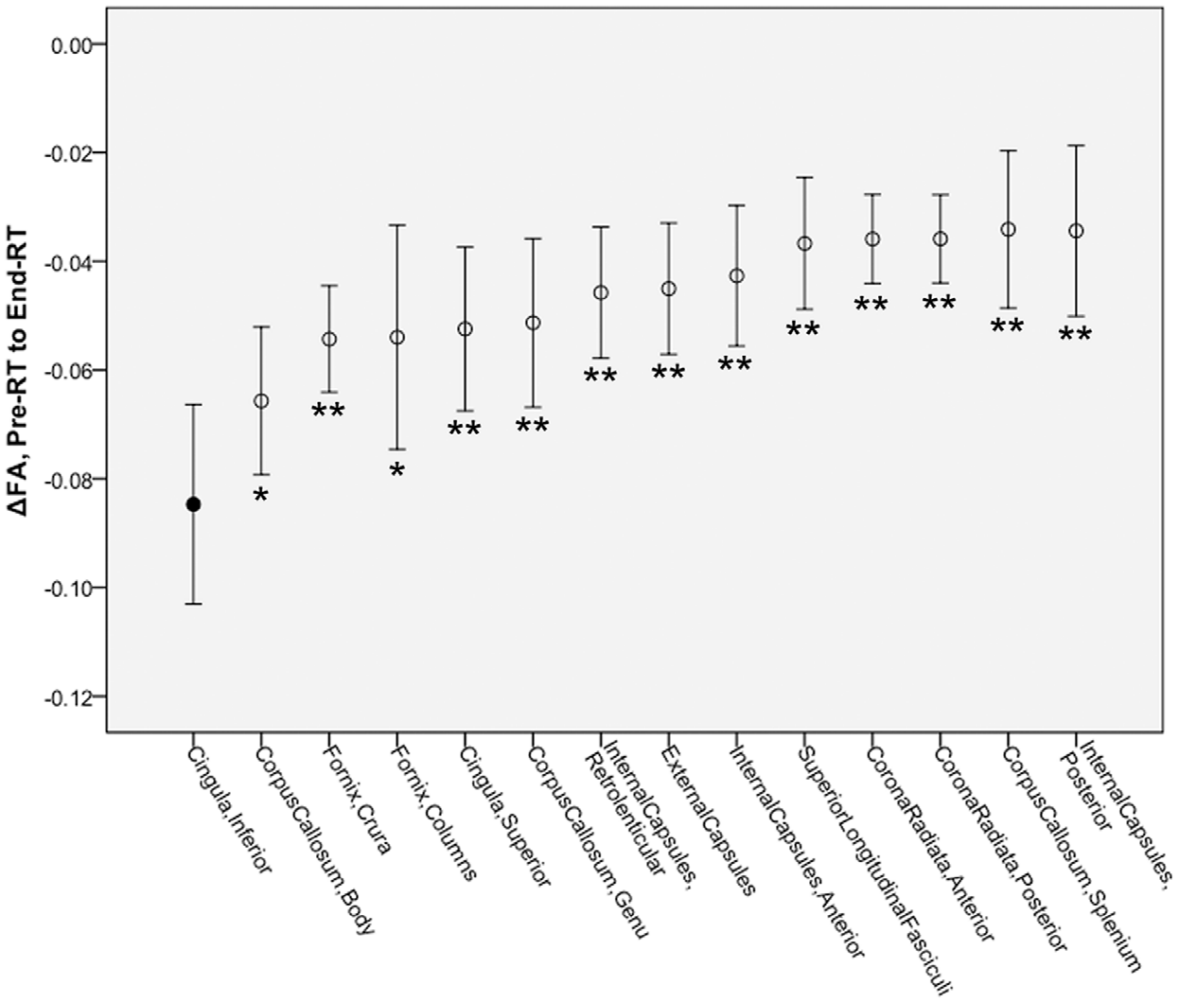
Mean FA changes from pre-RT to end-RT in fourteen structures. Comparisons by subject-paired two-sided *t*-test to Inferior Cingula (filled marker). *p<0.05; **p<0.00385 (Bonferroni correction). Error bars are 95% confidence interval.

Post hoc comparisons were performed between the structure with the greatest pre-RT to end-RT FA change (inferior cingula) and all other structures using subject-paired two-sided *t*-tests. All 13 comparisons had *p*<0.05, and 11 remained statistically significant after Bonferroni correction (*p*<0.00385). This indicates that the inferior cingula show greater FA decrease from pre-RT to end-RT than almost all other structures (Figure 6).

## Discussion

This study found (i) large regional variation in white matter diffusion index changes following radiation, representing varying susceptibility to treatment toxicity; and (ii) differences in the time course and spatial extent of changes in diffusion indices, implying specificity to distinct processes. Regional variation in diffusion index changes was greatest in fractional anisotropy (FA) and radial diffusivity (RD), and more extreme FA and RD changes were seen in inferior and superior cingula, the fornix columns and crura, and in sections of the corpus callosum. This likely represents greater vulnerability to the toxic effects of chemoradiotherapy in these structures, and may be related to the specific neurocognitive deficits seen following treatment. Temporal and spatial differences were seen between changes in RD and axial diffusivity (AD), demonstrating that that these diffusion indices may be specific for distinct pathologies and can be used to better examine white matter toxicity following chemoradiotherapy.

The inferior cingula and fornix showed large diffusion index changes, representing greater white matter degradation. These two structures are components of the limbic system, a functional circuit which also includes the hippocampus [39]. The limbic system as a whole functions to assist memory and learning and regulate emotional states and attention [40], processes commonly reported to be specifically impaired following brain radiation therapy [2]–[6]. This raises the hypothesis that selective sensitivity of the limbic circuit may be responsible for the specific pattern of functional impairments seen after chemoradiotherapy. This has previously been proposed, and could be tested by limbic circuit sparing during radiation therapy [21]. The present study also supports investigating diffusion tensor imaging of limbic circuit structures as a biomarker of radiation-induced neurocognitive impairment, just as reduced FA in the fornix and inferior cingula have been associated with other forms of neurocognitive impairment [41]–[43]. In a prior study, we found that neurocognitive tests after radiation were significantly correlated with diffusion index changes in the inferior cingula, but not in other temporal lobe white matter [26].

Changes in RD and AD occurred at different time intervals and locations. RD increases appeared to peak at the end of radiation, consistent with demyelination as the predominant early effect of radiation [12], [13], [28]. Fewer structures showed significant RD increases at one month after radiation, which may represent partial remyelination at this time point. Early demyelination and remyelination following brain irradiation has been observed in animal models [44]. By TBSS analysis, AD decreases appeared at one month after radiation, and there was virtually no spatial intersection between significant AD and RD changes. One possible explanation is that these reflect distinct pathologic processes, as has been seen in animal models [11]–[16]. Decreased AD could represent reactive astrogliosis and/or axonal degradation [11], [14]–[16]. Alternatively, decreased AD emerging in areas of previously increased RD could be due to resolving extracellular edema, which can affect both diffusion indices [10], [11]. However, this alternative is less plausible, as there was no edema detected by T2-weighted imaging. There was apparent left-right asymmetry in the occurrence of significant individual voxel changes, especially in AD (Figure 4). However, with direct comparison there was no significant asymmetry in mean diffusion index changes between paired structures. This could be due to within-structure variation, because we did not examine asymmetry in a voxel-wise manner. A previous study [18] also saw apparent asymmetry in diffusion index differences, but did not perform a direct comparison. At least one other study with direct comparison found no significant asymmetry in diffusion index changes after radiation [30].

The mechanism behind regional variation in white matter changes is still unclear. In addition to limbic circuit structures, large diffusion index changes were also seen in the corpus callosum and posterior superior longitudinal fasciculus, structures which like the limbic circuit are periventricular. These locations may be more susceptible to compressive damage from increased intracranial pressure, and a similar pattern has been seen with hydrocephalus [45]. However, increased intracranial pressure is not known to be a major effect of chemoradiotherapy in the absence of tumor progression or radiation necrosis. Alternatively, the deep location from the cortex means that these structures have lower capillary density and less blood flow [46], which has been associated with more severe white matter degradation after radiation [47]–[49]. A combination of mechanisms may be present, as animal models show early periventricular and vascular damage simultaneously [49], [50]. The addition of functional imaging techniques sensitive to vascular changes such as dynamic contrast enhanced MRI may help clarify this question [51].

One limitation of any imaging study is the potential for partial volume effect, i.e. values in the voxels of interest representing multiple tissue types. An advantage of tract based spatial statistics is that by identifying the tract center, voxels likely to contain partial volume effect at the periphery of the tract are excluded [31]. This remains a concern for small-caliber tracts, especially when the tract diameter approaches voxel size. For example, mean diameter of the fornix columns has been reported to be 2.3 mm [39], compared with our 1.75 mm original voxel size. This is reflected by the greater uncertainty in our estimates of diffusion index changes in the fornix columns, especially in AD and RD, due to partial volume effect from neighboring cerebrospinal fluid. Repeat imaging minimizes the impact of partial volume effect on our conclusions, as diffusion properties of cerebrospinal fluid are not expected to be systematically influenced by chemoradiotherapy. Additionally, tumor burden may still have affected our results despite no apparent tumor influence on the structures of interest by T1- and T2-weighted imaging. This was likely also minimized by repeat imaging allowing for within-patient compensation of tumor effects, and the fact that tumors were fairly evenly distributed throughout the brain across patients.

No interaction effect was found by age, sex or radiation dose on diffusion index changes, strengthening the validity of our results but also possibly reflecting our small sample size. A previous study has observed greater radiation-induced diffusion index changes in adults over sixty-five [30], however only two of our subjects were older than sixty-five years. A variation with radiation dose may have been expected, as previous studies have shown a dose-response relationship in diffusion index changes [26], [28]. However, this relationship was seen over larger ranges of 5 to 60 Gy. In this study, dose distribution between structures within a single patient was assumed to be uniform. Although this cannot be precisely true, opposed lateral field technique typically results in 97% of the brain receiving between 98% and 110% of the prescription dose [52]. We would not expect to see major differences in response within this range. The distribution of chemotherapy and primary cancer types in our study did not allow for statistically powerful comparisons between these groups. Bortezomib has been associated with peripheral neuropathy [53] and platinum-based agents such as carboplatin are associated with ototoxicity and peripheral neuropathy [54], however the short term effects of these agents on the central nervous system and possible interactions with radiation therapy are not known. A larger number of subjects, as well as longer follow up and neurocognitive testing could help determine the relationship between these factors and neurocognitive impairment.

### Conclusion

Our study demonstrates regional variation in white matter diffusion index changes following chemoradiotherapy, with larger changes implying greater white matter degradation. Large changes in the inferior cingula and fornix have implications for predicting and preventing treatment-induced neurocognitive impairment. We also address the specificity of diffusion indices and their potential utility for clarifying the mechanisms of white matter radiation injury.

## Supporting Information

Figure S1 Significant changes in FA from pre-RT to one month post-RT. Arrowheads: Z = −15, left inferior cingulum; Z = +25, corpus callosum body; Y = −40, left inferior cingulum; X = 0, fornix columns. Significant results per color chart, blue is TBSS skeleton without significant results. Depicted on MNI ICBM152 standard brain T1-weighted image [34].
(TIF)

Figure S2 Significant changes in RD from pre-RT to one month post-RT. Arrowheads: Z = +10, left fornix crus; Z = +25, corpus callosum body; Y = −40, left inferior cingulum; X = 0, fornix columns. Significant results per color chart, blue is TBSS skeleton without significant results. Depicted on MNI ICBM152 standard brain T1-weighted image [34]. Units are µm^2^/s.
(TIF)
