# Supplementary figures and images for: Regional Variation in Brain White Matter Diffusion Index Changes following Chemoradiotherapy: A Prospective Study Using Tract-Based Spatial Statistics
Source: PLoS One. 2013 Mar 4;8(3):e57768. doi: 10.1371/journal.pone.0057768 (PMC3587621; doi:10.1371/journal.pone.0057768)

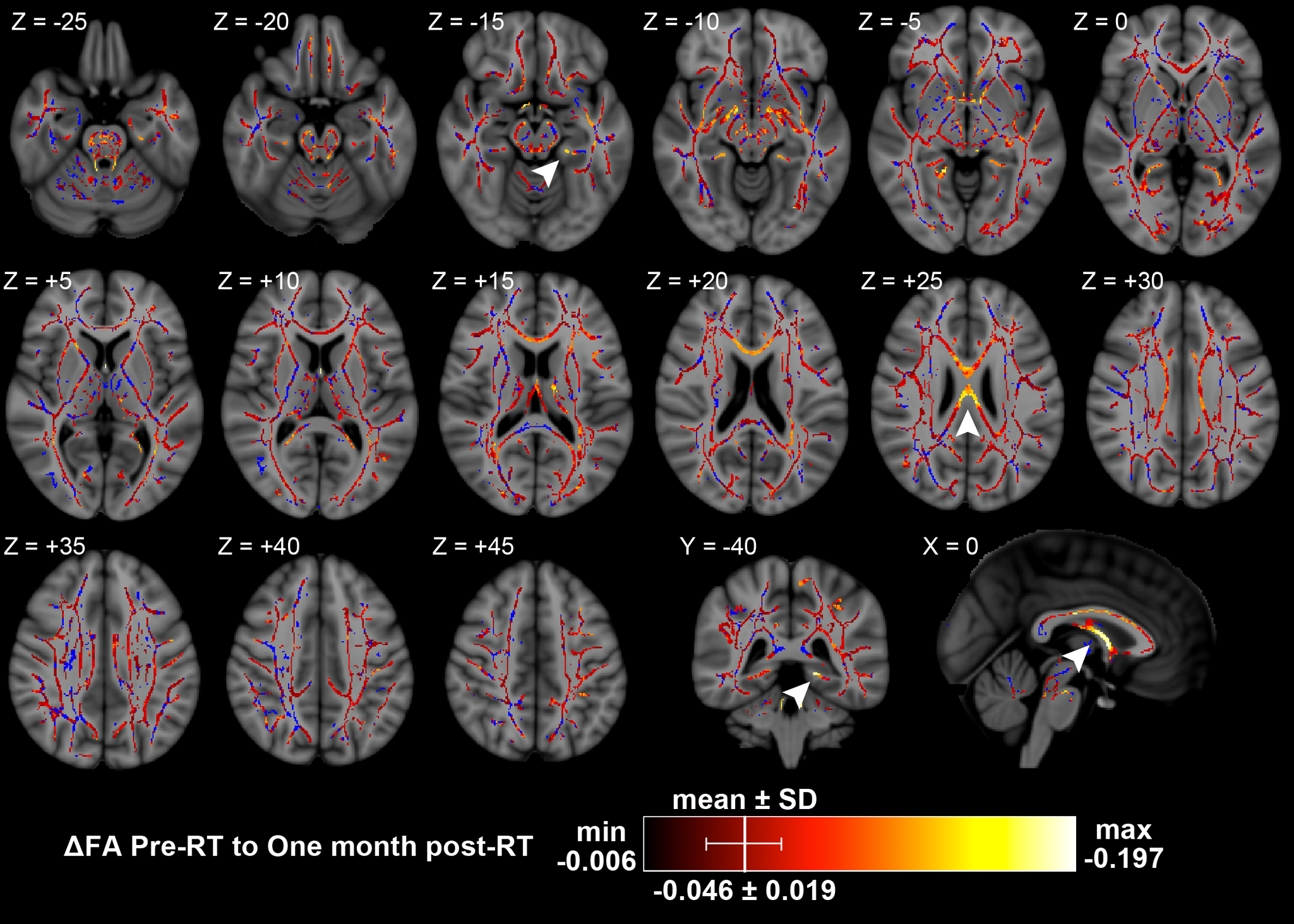

Supplement: Figure S1 — Significant changes in FA from pre-RT to one month post-RT. Arrowheads: Z = −15, left inferior cingulum; Z = +25, corpus callosum body; Y = −40, left inferior cingulum; X = 0, fornix columns. Significant results per color chart, blue is TBSS skeleton without significant results. Depicted on MNI ICBM152 standard brain T1-weighted image [34]. (TIF) [file pone.0057768.s001.tif]

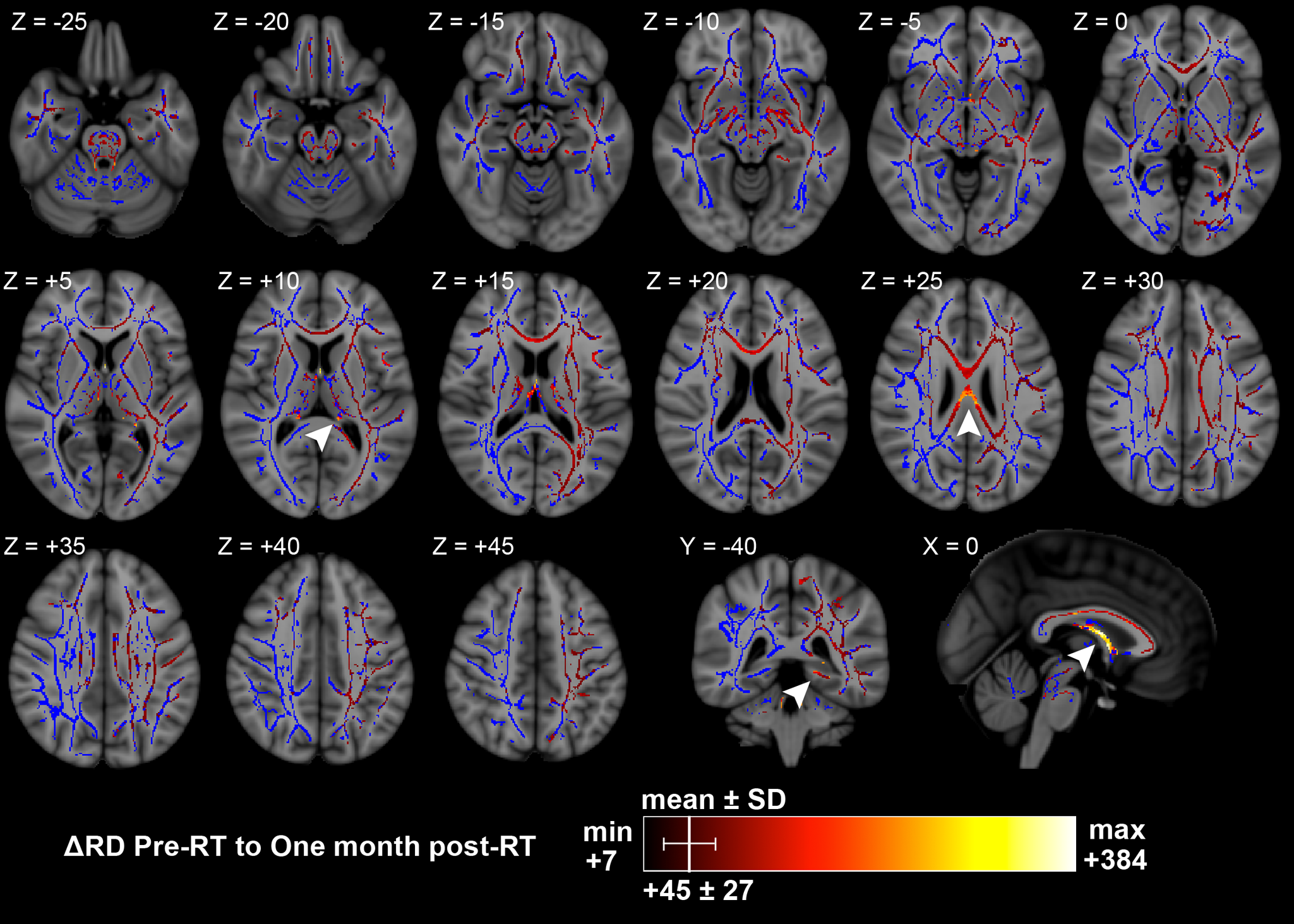

Supplement: Figure S2 — Significant changes in RD from pre-RT to one month post-RT. Arrowheads: Z = +10, left fornix crus; Z = +25, corpus callosum body; Y = −40, left inferior cingulum; X = 0, fornix columns. Significant results per color chart, blue is TBSS skeleton without significant results. Depicted on MNI ICBM152 standard brain T1-weighted image [34]. Units are µm2/s. (TIF) [file pone.0057768.s002.tif]
